# Genome Wide Analysis Indicates Genes for Basement Membrane and Cartilage Matrix Proteins as Candidates for Hip Dysplasia in Labrador Retrievers

**DOI:** 10.1371/journal.pone.0087735

**Published:** 2014-01-30

**Authors:** Ineke C. M. Lavrijsen, Peter A. J. Leegwater, Alan J. Martin, Stephen J. Harris, Marianna A. Tryfonidou, Henri C. M. Heuven, Herman A. W. Hazewinkel

**Affiliations:** 1 Department of Clinical Sciences of Companion Animal, Faculty of Veterinary Medicine, Utrecht University, Utrecht, The Netherlands; 2 Waltham Centre for Pet Nutrition, Leicestershire, United Kingdom; Harvard Medical School, United States of America

## Abstract

Hip dysplasia, an abnormal laxity of the hip joint, is seen in humans as well as dogs and is one of the most common skeletal disorders in dogs. Canine hip dysplasia is considered multifactorial and polygenic, and a variety of chromosomal regions have been associated with the disorder. We performed a genome-wide association study in Dutch Labrador Retrievers, comparing data of nearly 18,000 single nucleotide polymorphisms (SNPs) in 48 cases and 30 controls using two different statistical methods. An individual SNP analysis based on comparison of allele frequencies with a χ^2^ statistic was used, as well as a simultaneous SNP analysis based on Bayesian variable selection. Significant association with canine hip dysplasia was observed on chromosome 8, as well as suggestive association on chromosomes 1, 5, 15, 20, 25 and 32. Next-generation DNA sequencing of the exons of genes of seven regions identified multiple associated alleles on chromosome 1, 5, 8, 20, 25 and 32 (p<0.001). Candidate genes located in the associated regions on chromosomes 1, 8 and 25 included *LAMA2*, *LRR1* and *COL6A3*, respectively. The associated region on CFA20 contained candidate genes *GDF15*, *COMP* and *CILP2*. In conclusion, our study identified candidate genes that might affect susceptibility to canine hip dysplasia. These genes are involved in hypertrophic differentiation of chondrocytes and extracellular matrix integrity of basement membrane and cartilage. The functions of the genes are in agreement with the notion that disruptions in endochondral bone formation in combination with soft tissue defects are involved in the etiology of hip dysplasia.

## Introduction

Hip dysplasia is characterised by an abnormal formation of the hip joint, causing incongruity and/or laxity of the joint, which can lead to osteoarthritis. It has been observed in several mammals, including humans, where it is referred to as developmental dysplasia of the hip (DDH), and in dogs where the term canine hip dysplasia (CHD) is used. In both species delayed femoral capital ossification, hip joint laxity and subluxation are observed in dysplastic hips [1] [2].

The reported incidence of DDH varies widely, depending on screening methods and DDH definition employed at medical centres. Between 0.5% and 4.2% of screened neonates and infants receive treatment [2] [3]. Risk factors include a family history of DDH, female sex, other skeletal abnormalities and hormonal and environmental factors. CHD is one of the most common developmental skeletal disorders in dogs and affects predominantly breeds of medium and large sized dogs [4]. In Bulldogs up to 72% of the screened animals are affected (www.offa.org/stats_hip.html). For some breeds a sex predisposition has been described [5]. Hereditary and environmental factors are thought to play a role in the manifestation of this disorder. The involvement of one gene with a major effect in combination with genes with small contributions is considered likely in a number of breeds [6] [7].

In recent years, several attempts have been made to gain understanding of the molecular genetic basis of hip dysplasia in man and dog. Occurrence of hip dysplasia in humans has been associated with variations in asporin (an extracellular matrix protein belonging to the small leucine-rich repeat protein family), interleukin-6 and transforming growth factor β1 [8] (both involved in bone remodelling [9] [10]), growth differentiation factor 5 [11], T-box 4 (involved in hind limb development) [12], the vitamin D receptor and estrogen receptor 1 [13] and pregnancy-associated plasma protein A2 [14]. In canines, genome-wide studies have been performed in Portuguese Water Dogs, German Shepherd Dogs, Labrador Retrievers and Greyhound/Labrador Retriever crossbreeds. Different definitions of CHD were used, including hip joint laxity measured as Norberg Angle or distraction index and hip scores according to the Fédération Cynologique Internationale (FCI) for CHD grading. These studies found quantitative trait loci (QTLs) on multiple chromosomal regions and a variant of the fibrillin 2 gene (*FBN2*) to be associated with CHD [15]–[21]. None of the loci could account for more than 18% of the genetic variance, confirming the polygenic nature of the disorder. Additional genes play an essential role in the correct formation of the hip joint, and identification of these genes would provide insight into the molecular processes that lead to CHD.

We describe a SNP-based genome-wide association study for CHD in Labrador Retrievers from The Netherlands, and subsequent fine mapping of the identified regions by case-control comparison of DNA sequences of gene exons included in the regions.

## Methods

### Ethics Statement

The dogs were privately owned and included with informed consent of the owners. They were handled by licensed veterinarians only. Thus we complied to the conditions set forth in the Dutch ‘Wet op de Uitoefening van de Diergeneeskunde’ (Law on the Practice of Veterinary Medicine) of March 21, 1990 and approval of an ethics committee for the use of samples of the animals was not necessary.

### Animals

Hip radiographs and DNA from blood samples of 122 unrelated purebred Labrador retrievers born between 1980 and 2005 were assessed. All dogs were registered by the Dutch Kennel Club or descendants of registered dogs only. DNA was isolated from blood samples using a standard salt-extraction method [22]. Blood samples were not available from 26 dogs and the DNA was isolated from buccal swabs from these dogs using the QIAamp DNA Blood Mini Kit (QIAgen, Hilden, Germany).

### Phenotypes

A ventrodorsal hip radiograph of the dog in dorsal recumbency with extended hind limbs was made, examined by the CHD-panel of the Dutch Kennel Club and graded according to FCI guidelines, distinguishing CHD-A (normal), CHD-B (subnormal/borderline), CHD-C (mild), CHD-D (moderate) and CHD-E (severe). Most dogs were graded at age between 12 and 20 months. The six exceptions were one CHD-C graded dog of 50 months; three CHD-D dogs of 26, 27 and 40 months; and two CHD-D/E graded dogs of 33 and 63 months of age. We chose to analyze canine hip dysplasia as a binomial trait with affected (CHD-C/D/E) versus unaffected (CHD-A) dogs excluding CHD-B dogs from the study.

### Generation of Genotypes and Quality Control

The Illumina Infinium CanineSNP20 BeadChip was used to genotype more than 22,000 SNPs in our sample set. Of these, the 17,859 SNPs that had a minor allele frequency higher than 0.01 and that were successfully genotyped in more than 90% of the samples were included in the data analysis. PLINK software was used to generate an “identical by state” similarity matrix [44]. The first two principal components of this multidimensional similarity matrix were used to depict each individual in a 2-dimensional plot.

### Association Analysis

A χ^2^ statistic for the comparison of allele frequencies of 17,859 SNPs in 48 cases (21 males and 27 females) and 30 unaffecteds (12 males and 18 females) was generated using PLINK software [23]. Of the cases, 17 were graded CHD-C, 25 CHD-D and 6 CHD-D/E. The unaffecteds were all graded CHD-A.

A Bonferroni correction was used as multiple-testing correction, but because this is a rather stringent correction method, a series of 1,000 permutations were also run on our data set and compared against the best result from all SNPs to estimate the significance of the peaks (EMP2 in PLINK).

Simultaneous analysis of multiple randomly selected SNPs was performed using a Bayesian procedure with the program iBay [24] [25] assuming a mixture model to identify associated SNPs. The model assumed that SNPs belong to one of two normal distributions; the first distribution contained SNPs that had very little effect on the phenotype and the second distribution comprised SNPs that had an effect on the phenotype. Bayesian analysis required values for the hyper parameters of the priors. It was assumed that less than 5% of our SNPs belonged to the second distribution explaining 99.5% of the genetic variance.

The genetic variance was obtained from the phenotypic variance assuming a heritability of 20%. This value was an average of the heritability derived for four dog breeds (unpublished results). The prior variances of the two distributions were calculated using these assumptions. The Bayesian procedure is based on Gibbs sampling and three million Bayesian iterations were run to calculate the posterior variances as well as the posterior probabilities that SNPs belong to either of the two distributions. Odds ratios were calculated for each SNP by dividing the posterior probability by the a priori probability, which was inherent to the assumptions made. A Bayes factor BF was calculated by dividing the posterior by the prior odds ratio for each SNP. BF larger than 3.2 indicate ‘substantial’ evidence for association while a BF larger than 10 indicates ‘strong’ evidence and a value larger than 100 as ‘decisive’ [26]. We considered SNPs with a BF>3 as regions for follow up.

### Next-Generation Sequencing and Analysis

Five regions were selected for targeted exon enrichment and sequencing on a SOLiD4 at the University of Liverpool, UK. For each annotated gene in these regions, exons, intron/exon boundaries (30 bp) and the intergenic regions flanking both sides of each gene (2×500 bp), were targeted for enrichment. When the gene was smaller than 3 kb, the 5′ (upstream) flanking region was increased until this criterion was met, to facilitate haplotype analysis. To minimize the chance of missing genes or exons due to a faulty annotation of the dog genome build, the synthenic regions in the human genome were compared and human cDNA of genes in these regions were compared with the reference dog genome using BLAST software. When the orientation of the alignments was corresponding to the homologous gene in the dog and located within 100 kb of the gene, this alignment was considered a putative non-annotated exon. These exons, as well as alignments of canine expressed sequence tags (ESTs) compared with the dog genome were targeted for enrichment. Microarray-based enrichment of all targeted loci was performed using a custom Comparative Genomic Hybridization Array by Roche NimbleGen.

A subset of “interesting” genes, based on their known function and position within the association peak regions, was selected and the exons were sequenced in 48 cases and 30 controls (priority genes), while the exons of remainder of the genes were sequenced in 15 cases and 15 controls (non priority genes).

Minor allele read frequencies for each sequenced location were calculated by dividing the number of observed reads for the less prevalent allele by the number of the reads that covered the position of the SNP. Genotypes were estimated using thresholds for minor allele read frequencies of more than 0.2 and less than 0.8 for heterozygotes. Individuals with a minor allele read frequency of less than 0.2 at a certain location were considered homozygous for the major allele, and over 0.8 were considered homozygous for the minor allele. These inferred genotypes were used to calculate allelic association with a standard χ2 statistic as well as with iBay. All raw SNP data and DNA sequencing data are available upon request.

## Results

Population stratification analysis revealed genetic divergence within the group of Labrador Retrievers. The Dutch Labrador Retrievers were identified as the main group, and samples that deviated from this group were dogs imported from the USA or their first generation descendants (n = 44). There were no cases in this subpopulation and we excluded this group, leaving 48 cases (mild, moderate or severe CHD) and 30 controls (no signs of CHD) for further analysis.

### Individual and Multiple SNP Association Analysis

**Figure 1:**
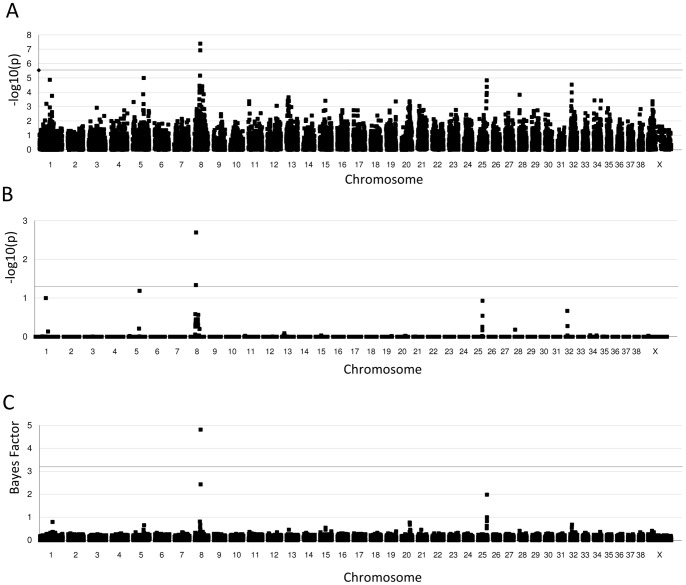
Genome wide association analysis of hip dysplasia in Labrador retrievers. Genotypes of 17,859 SNPs were compared between 48 cases and 30 controls. A. Allele frequency based χ^2^ statistics. The dotted line indicates the significance threshold after Bonferroni correction. B. Multiple testing correction of the χ^2^ statistics by 1,000 permutations of the phenotypes to determine empirical p-values. The dotted line indicates the significance threshold (α = 0.05). C. Genome wide association analysis using Bayesian variable selection to detect SNPs with a high probability to have an effect on the CHD phenotype. The dotted line indicates the significance level.

Individual SNP association to CHD was tested using a χ^2^-based statistic for the comparison of allele frequencies using PLINK software, and revealed association to multiple chromosomal regions, including chromosome 1, 3, 5, 8, 11, 12, 13, 15, 19, 20, 25, 28, 32, 34 and the X chromosome (P-value <0.001, Figure 1). A Bonferroni correction of α = 0.05 over 17,859 tests was performed to correct for multiple testing and only the region on chromosome 8 was significantly associated to CHD, with BICF2S23913508 located at position 33707642 bp of the reference genome CanFam2 as the most strongly associated SNP in this region (corrected P-value = 0.0007, Figure 1A). Permutations were performed as a less stringent correction for multiple testing, but it also resulted in significant association of the region on chromosome 8 only (corrected *P*-value = 0.002, Figure 1B).

A Bayesian variable selection method was used to study the combined effect of all SNPs simultaneously to distinguish between chromosomal regions likely to have an effect on the phenotype and false positives. The effect of a SNP is corrected for all other SNPs in the model. The underlying assumption in this simultaneous SNP association analysis is that SNPs belong to either of two distributions; one that contains SNPs that contribute to the phenotype, and one that contains SNPs that do not. The program iBay was used to generate posterior probabilities, expressed as Bayesian factors (BF), for each SNP to belong to the distribution of SNPs that had an effect on the phenotype. Bayesian factors larger than three were considered significant. Analysis of the SNP data of the 48 cases and 30 controls identified one genomic region located on chromosome 8 to have an effect on the phenotype with BF greater than 3. Additional regions were identified that have a minor contribution (BF>0.5) to the phenotype on chromosomes 1, 5, 15, 20, 25 and 32 (Figure 1C). In Table 1 the results of the 18 SNPs with a BF larger than 0.5 are summarized. At least one SNP in each of the seven regions passed this threshold. The strongest association with the highest number of SNPs and the highest BFs were observed on chromosome 8 and on chromosome 25 and only eight SNPs were identified in the other five regions together. Regions nominated for follow-up analysis were selected based on the individual and multiple SNP analyses, and included regions on CFA01 (70.7–71.9 Mb), CFA05 (58.6–63.5 Mb), CFA08 (28.0–34.5 Mb), CFA15 (32.2–33.6 Mb), CFA20 (46.3–51.2 Mb), CFA25 (47.1–51.9 Mb), CFA32 (11.2–13.0 Mb).

**Table 1:** Array SNPs associated with hip dysplasia in Labrador Retrievers ^a^.

			MAF^b^		score	
CFA	location	SNP	cases	controls	PLINK^c^	iBay^d^
1	70962581	BICF2P1446667	0.24	0.02	3.76	0.79
5	62363747	BICF2S23057444	0.22	0.57	5.02	0.66
8	29868871	BICF2P950415	0.28	0.62	4.47	0.81
8	31218201	BICF2S23118415	0.46	0.15	4.12	0.57
8	32367889	BICF2S2305197	0.56	0.23	4.26	0.68
8	33707642	BICF2S23913508	0.20	0.63	7.41	2.43
8	33990753	BICF2P601580	0.64	0.20	6.94	4.82
15	32865368	BICF2G630433123	0.28	0.57	3.42	0.54
20	46604376	BICF2P1445357	0.40	0.68	3.32	0.77
20	47759323	BICF2S22943564	0.40	0.67	3.00	0.68
20	47926376	BICF2P878084	0.34	0.63	3.39	0.75
25	50228063	BICF2G630160005	0.54	0.23	3.83	0.63
25	50458146	BICF2S23326254	0.28	0.03	3.97	0.52
25	50637313	BICF2G630159246	0.55	0.20	4.85	1.98
25	51127510	BICF2P909436	0.53	0.20	4.39	1.00
25	51155516	BICF2P770517	0.53	0.20	4.39	0.84
32	11802242	BICF2S23211582	0.07	0.33	4.54	0.54
32	12336449	rs8795055	0.20	0.47	3.43	0.68

a SNPs with a Bayes Factor >0.5 were included.

b MAF = minor allele frequency.

c –^10^log p-values.

d Bayes Factor.

### Targeted Fragment Enrichment and Next-Generation Sequencing

The seven regions that displayed association were selected for microarray-based enrichment and DNA sequencing. The total size of these 7 regions was ∼25 Mb. An exon sequencing approach was chosen to increase the coverage per analyzed position, resulting in 2.3 Mb of target DNA. Priority genes in the regions were selected based on the function of the encoded proteins. The processes involved were cartilage or bone development and synthesis or maintenance of extracellur matrix. The priority gene exons totaled 300 kb and these were analyzed in 69 DNA samples from the Dutch Labrador Retriever population. The exons of the remaining non-priority genes, 2 Mb in total size, were enriched and sequenced in 15 cases and 15 controls. For both sets, approximately 30% of the generated reads could be mapped to the targeted regions.

In the priority samples, approximately 81% of the targeted regions were sequenced at least once, but only 65% of the targeted regions were covered 10 times or more. For the non-priority samples, these percentages were slightly lower (80% and 60%, respectively) per sample. Overall, 74.4% of the priority regions and 58.0% of the non-priority regions were sequenced at least 10 times in 10 cases and 10 controls. We identified 5388 variations that were covered at least 10 times in both cases and controls and that had a minor allele frequency higher than 0.01 (Table S1). Genotypes were inferred from the number of reads per allele and used to calculate χ^2^-based allelic association. The SNPs that were most strongly associated with CHD are listed in Table 2 together with the genes at the position or in the vicinity of these SNPs.

**Table 2:** Variant DNA sequences associated with hip dysplasia in Labrador Retrievers.

			number		MAF a					
CFA	location	alleles	cases	controls	cases	controls	score b	gene	position in gene c	
1	70938018	(T/A)	36	30	0.28	0.02	4.35	*LAMA2*	intron	
1	70997779	(A/T)	33	30	0.26	0.02	3.94	*LAMA2*	exon, synonymous	
5	59194609	(G/−)	15	15	0.33	0.77	3.13	*KLHL17*	3′-UTR	
5	62929475	(C/T)	14	8	0.04	0.63	4.85	*NPHP4*	5′-UTR	
8	29247021	(C/T)	33	30	0.73	0.35	4.67	*LRR1*	3′-UTR	
8	31496895	(C/T)	33	30	0.55	0.23	3.46	*PTGDR*	downstream	
8	31496910	(C/T)	32	30	0.53	0.22	3.51	*PTGDR*	downstream	
20	45260332	(A/G)	32	30	0.30	0.07	3.01	*LTF*	exon, non-synonymous	
20	46671813	(C/G)	15	15	0.53	0.10	3.51	*PBX4*	inton	
20	46706734	(A/G)	27	28	0.63	0.27	3.87	*CILP2*	3′-UTR	
20	47636184	(G/A)	29	30	0.55	0.23	3.41	*GDF15*	3′-UTR	
20	47714388	(G/A)	29	29	0.24	0.66	5.13	*LSM4*	5′-UTR	
20	48071192	(G/A)	34	30	0.26	0.63	4.56	*INSL3*	upstream	
20	48243399	(G/−)	12	13	0.17	0.65	3.31	*UNC13A*	3′-UTR	
20	48804130	(G/A)	13	14	0.19	0.71	3.92	*NWD1*	exon, synonymous	
20	49019261	(T/C)	29	30	0.57	0.25	3.38	*CHERP*	intron	
20	49318725	(C/T)	7	12	0.64	0.04	4.31	*CIB3*	exon, synonymous	
25	47629272	(C/T)	26	29	0.06	0.36	3.94	*INPP5D*	upstream	
25	47666842	(G/A)	28	30	0.02	0.28	4.10	*INPP5D*	exon, synonymous	
25	47685188	(C/T)	31	30	0.68	0.37	3.23	*INPP5D*	intron	
25	48075297	(C/G)	15	15	0.37	0.00	3.62	*LOC100688622*	exon, non-synonymous	
25	48076278	(G/A)	15	15	0.37	0.00	3.62	*LOC100688622*	downstream	
25	48396330	(C/T)	25	30	0.54	0.23	3.03	*SPP2*	inron	
25	50050076	(G/A)	9	6	0.78	0.08	3.71	*ASB18*	downstream	
25	50228063	(C/G)	13	15	0.27	0.73	3.28	*IQCA1*	intron	
25	51029326	(G/A)	28	30	0.57	0.27	3.06	*COL6A3*	intron	
25	51031100	(A/G)	32	30	0.39	0.10	3.73	*COL6A3*	exon, synonymous	
25	51040259	(A/G)	33	30	0.52	0.20	3.61	*COL6A3*	exon, synonymous	
25	51046607	(G/A)	31	30	0.53	0.22	3.49	*COL6A3*	exon, synonymous	
25	51736576	(T/C)	27	30	0.56	0.23	3.38	*HES6*	upstream	
32	11265116	(−/T)	15	15	0.03	0.40	3.25	*LOC487839*	downstream	

a minor allele frequency.

b –^10^log p-value.

c UTR lengths based on human cDNA data.

## Discussion

CHD is considered a complex trait and multiple chromosomal regions have been associated with the disease in various dog breeds [15]–[20]. Around the world different protocols are in use to classify the severity of the disease based on radiographs. In this study, the severity grade was not taken into account due to the limited number of samples and CHD was analysed as a binomial trait. Given that HD is a complex disease two statistical analyses were applied. In contrast to the single SNP-analysis with PLINK software, the multiple-SNP analysis of iBay takes this complexity into account and therefore more weight was given to the results obtained with the latter program.

As could be expected for a complex disorder, our genome-wide analysis revealed multiple regions associated with CHD. Several of these regions had been implicated in CHD before. The region on CFA08 at position 28–34.5 Mb that was strongly associated in our study overlaps largely with the region of 29–34 Mb found by Marshall and Distl [16] in the German Shepherd Dog. The less strongly associated region on CFA01 at 70.7–71.9 Mb overlaps with a region around position70 Mb identified by Phavaphutanon et al. [18] in Labrador Retrievers and the region on chromosome 20 at 46.3–51.2 Mb overlaps with the region of 40–47 Mb described by Todhunter et al. [20] in the offspring of affected Labrador Retrievers and unaffected Greyhounds. The minor associated region on CFA32 at 11.2–13.0 Mb is flanked by regions identified in Labrador Retrievers around 5 Mb [18] and German Shepherd Dogs (16–20 Mb) [16].

We found no evidence for association of other regions on chromosomes 1, 2, 10, 20 (two regions around 30 Mb and 60 Mb) and CFA22 previously reported to be associated in Labrador Retrievers [18]. We also did not observe association to the region of CFA11 that contains *FBN2* coding for fibrillin 2. A deletion in an intron of this gene was found to be associated with suppression of the gene and with hip dysplasia in Labrador Retrievers from the USA [21]. The discrepant results might be due to population differences. Indeed, the population stratification analysis indicated that the Dutch and American populations of Labrador Retrievers have diverged.

CHD is a common disorder occurring in many breeds, and alternate genetic factors may be involved in different breeds or even populations. The etiology of CHD is not understood, although two broad etiological categories have been proposed i.e., laxity of the peri-articular soft tissues (ligaments, muscles, joint capsule) [27]–[30], and an abnormal progression of the endochondral ossification in the hip joint, or a combination of both processes [31] [32]. A majority of 88% of the affected dogs in our sample was graded before the age of 20 months. We were not concerned with the possibility of including phenocopies by adding dogs that were diagnosed at a later age, because their number was small and these could not have led to falsely positive results.

One of the identified SNPs associated with CHD is located near the *LRR1* gene on CFA08. the encoded protein has been described as down regulating the 4-1BB-mediated signal transduction pathways JNK1 and NfkappaB [33]. JNK1 and NFkappaB play a role in proteoglycan synthesis by chondrocytes [34] [35] making a role for *LRR1* in cartilage development and physiology plausible. Functional studies are needed to corroborate the possible role of *LRR1* in CHD.

Additional analysis of the data resulted in the selection of other SNPs of interest that were associated with CHD status together with *LRR1*. These SNPs were located in the top regions of the multiple SNP association analysis in or near the genes *COL6A3* on CFA25, and *LAMA2* on CFA01 (Table 2). The specific function of these genes in cartilage and muscle as described below facilitates their implication in CHD. First, *COL6A3* codes for the alpha 3 chain of collagen VI that forms a fine fibrillar network in many connective tissues [36] and is a specific component of the peri-cellular matrix of chondrocytes with a protective role for these cells [37]. Furthermore, one of the characterising features of myopathies related to mutations in the *COL6A3* gene is joint hyper laxity [38]. Second, laminins with a *LAMA2* encoded chain are found in the basal lamina of basement membranes [39]; the chain is also related to cartilage development [40] and has been implicated in congenital muscular dystrophy [41]. Third, the region of interest on CFA20 contains three genes involved in connective tissue integrity. This region displays strong linkage disequilibrium and it is not possible to discriminate between the genes based on the association data. Good candidate genes for involvement in CHD in this region are *GDF15* encoding growth-differentiating factor 15, *COMP* for cartilage oligomeric matrix protein and the gene encoding cartilage intermediate layer protein 2 (*CILP2*). *GDF15* is a gene with a role not only in bone remodelling [42], but has also been related to osteoarthritis in humans [43]. Mutations in *COMP* are associated with pseudoachondroplasia (OMIM177170) and multiple epiphyseal dysplasia (OMIM132400). *CILP-2* is expressed in cartilaginous tissues and muscles; specifically there are indications that it is associated with collagen VI microfibrils mediating interactions between matrix components in cartilage [44]. It should be noted that our DNA sequencing strategy aimed at identification of mutations in exons and in intron/exon junctions. Intron mutations at positions not close to exons can affect RNA splicing and these were not captured by our strategy.

The associated regions support the view that cartilage, as a specified connective tissue, plays an important role in the pathophysiology of CHD. A delay in endochondral ossification of the femoral head has been demonstrated in dysplastic Labrador dogs [45]–[47], in correspondence with earlier findings in German Shepherd dogs [28]. Furthermore, CHD in American Labrador Retrievers is associated with a deletion in *FBN2* that codes for fibrillin2, a component of the extracellular matrix in fibrous joint capsule and articular cartilage [21]. Another study observed a difference in muscle composition of two months old German Shepherd Dogs that developed CHD later in life compared to controls [28]. In addition, structural alterations were found in collagen fibers of the joint capsule of the hip joint and of the round ligament of dysplastic Labrador Retrievers when compared to these structures in tight-hipped Labradors [1].

## Conclusions

This study identified several SNPs associated with CHD in or near genes that are involved in extracellular matrix development. The combination of the candidate genes implicates bone and soft tissue development in CHD. First, disturbances during the process of endochondral bone formation attributing to the abnormal formation of the hip joint may well be related to disturbances in the hypertrophic differentiation of the chondrocytes. Second, there are indications for soft tissue involvement (cartilage, muscles, and ligaments). The association results described here need to be confirmed by analysis of an independent replication cohort. Additional mechanistic studies are essential for the understanding of the molecular processes that lead to hip dysplasia.

## Supporting Information

Table S1 Observed DNA sequence variations in genome regions associated with canine hip dysplasia.
(XLS)
